# *Bacillus subtilis* Matrix Protein TasA is Interfacially Active, but BslA Dominates Interfacial Film Properties

**DOI:** 10.1021/acs.langmuir.3c03163

**Published:** 2024-02-14

**Authors:** Ryan J. Morris, Natalie C. Bamford, Keith M. Bromley, Elliot Erskine, Nicola R. Stanley-Wall, Cait E. MacPhee

**Affiliations:** † School of Physics & Astronomy, University of Edinburgh, Peter Guthrie Tait Road, Edinburgh EH9 3FD, U.K.; ‡ National Biofilms Innovation Centre, Southampton SO17 1GB, U.K.; § Division of Molecular Microbiology, School of Life Sciences, University of Dundee, Dundee DD1 5EH, U.K.

## Abstract

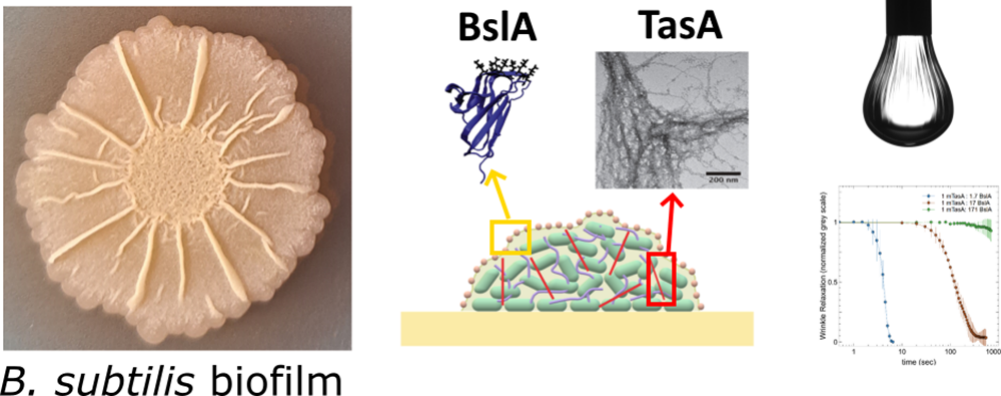

Microbial growth often occurs within multicellular communities called biofilms, where cells are enveloped by a protective extracellular matrix. *Bacillus subtilis* serves as a model organism for biofilm research and produces two crucial secreted proteins, BslA and TasA, vital for biofilm matrix formation. BslA exhibits surface-active properties, spontaneously self-assembling at hydrophobic/hydrophilic interfaces to form an elastic protein film, which renders *B. subtilis* biofilm surfaces water-repellent. TasA is traditionally considered a fiber-forming protein with multiple matrix-related functions. In our current study, we investigate whether TasA also possesses interfacial properties and whether it has any impact on BslA’s ability to form an interfacial protein film. Our research demonstrates that TasA indeed exhibits interfacial activity, partitioning to hydrophobic/hydrophilic interfaces, stabilizing emulsions, and forming an interfacial protein film. Interestingly, TasA undergoes interface-induced restructuring similar to BslA, showing an increase in β-strand secondary structure. Unlike BslA, TasA rapidly reaches the interface and forms nonelastic films that rapidly relax under pressure. Through mixed protein pendant drop experiments, we assess the influence of TasA on BslA film formation, revealing that TasA and other surface-active molecules can compete for interface space, potentially preventing BslA from forming a stable elastic film. This raises a critical question: how does BslA self-assemble to form the hydrophobic “raincoat” observed in biofilms in the presence of other potentially surface-active species? We propose a model wherein surface-active molecules, including TasA, initially compete with BslA for interface space. However, under lateral compression or pressure, BslA retains its position, expelling other molecules into the bulk. This resilience at the interface may result from structural rearrangements and lateral interactions between BslA subunits. This combined mechanism likely explains BslA’s role in forming a stable film integral to *B. subtilis* biofilm hydrophobicity.

## Introduction

**Figure 1:**
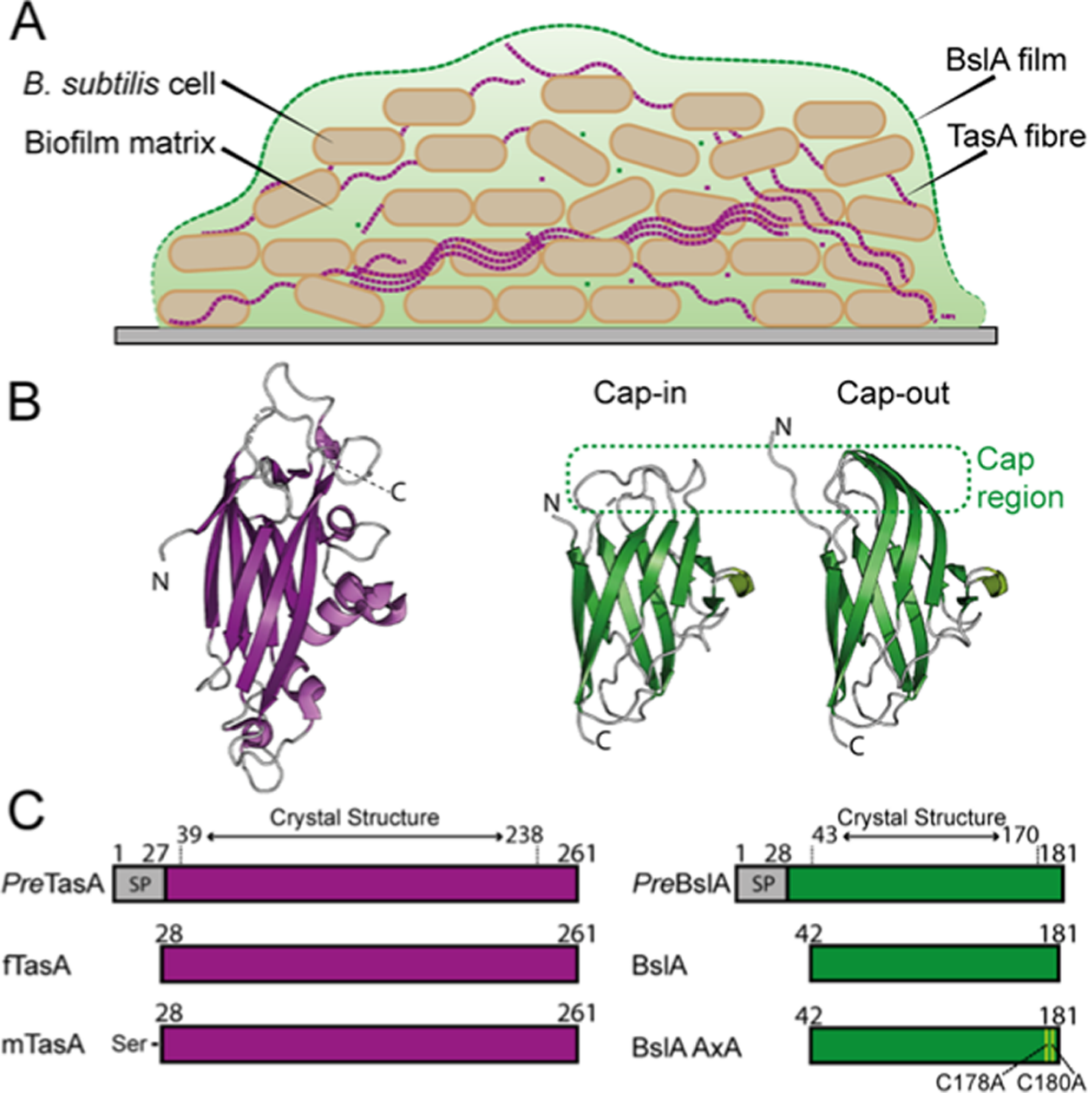
TasA and BslA are *B. subtilis* matrix proteins. (A) Schematic representation of a *B. subtilis* biofilm showing that the extracellular matrix surrounding the cells confers protection from environmental pressures. The matrix proteins TasA (purple) and BslA (green) are both secreted as monomers and can take on higher-order structures. The hydrophobic BslA film coats the colony biofilm and the TasA fibers contribute to structure and biofilm formation. The representation is for illustrative purposes and not to scale. (B) Cartoon representations of the crystal structures of TasA (purple, PDB 5OF2) and BslA (green, PDB 4HBU chains J [cap-in] and H [cap-out]). The N and C-termini are labeled with the appropriate letters. (C) Diagrams of the protein domains of TasA and BslA numbered based on the amino acid sequences of each protein. The unprocessed proteins (*Pre*TasA and *Pre*BslA) are displayed with signal peptides (SP) in gray and the secreted domains in purple or green. The recombinant constructs (fTasA, mTasA, BslA, and BslA AxA) are also shown for clarity.

Microbial biofilms are complex communities encapsulated in a self-produced matrix comprising proteins, carbohydrates, small metabolites, and extracellular DNA. The biofilm mode of growth confers many benefits to inhabitants such as increased resistance to environmental assault and change.^1,2^ The matrix components are diverse and dependent on the microbial species as well as environmental conditions.^2,3^ Some biofilm matrices have been found to include proteins with interesting properties. *Bacillus subtilis* is a model organism in the study of biofilm formation. The matrix produced by *B. subtilis* includes two key proteins: the interfacially active protein BslA, and the fiber-forming protein TasA (Figure 1A).^4−6^

BslA is a secreted protein exhibiting an immunoglobulin-fold (Figure 1B,C)^3^ that is required for *B. subtilis* sliding motility and biofilm structure.^4,7−9^ At a hydrophobic/hydrophilic interface, the “cap” of BslA (constituted by the loops at one end of the β-sandwich) undergoes a structural rearrangement, exposing hydrophobic residues to the hydrophobic phase. This rearrangement causes the loops that had no secondary structure to form a new β-sheet. The increase in β-sheet structure has been seen in both the crystal structure and circular dichroism spectroscopy of BslA-stabilized refractive index matched emulsions (RIMEs).^10^ This structural change has been predicted to create an energy barrier that prevents BslA from returning to the hydrophilic phase, thereby contributing to film stability.^11,12^ The protein then self-assembles laterally to form a regular lattice.^10,13^ Due to a cysteine motif (C178 and C180) at the C-terminus of BslA, the protein is found predominantly as a dimer in solution with the cap of one monomer adsorbing to the interface.^10^ Mutation of the cysteine motif to alanine residues produces a monomeric form of BslA (BslA AxA) that produces films of equivalent stability to the wildtype protein^14^ (Figure 1C). The ability of BslA to form stable elastic films at air/water and oil/water interfaces has been of interest to biotechnology, including formulation and food industries.^12,15−18^

TasA is the other main protein component of the *B. subtilis* matrix. Like BslA, TasA is a secreted protein with a β-sandwich fold^19^ (Figure 1B). Upon secretion, the signal peptide is cleaved by SipW, and the mature protein comprising residues 28 to 261 is released.^20^ The new N-terminus can participate in strand exchange with another TasA monomer, leading to polymerization of the monomers into filaments.^21^ The presence of TasA filaments, or fibers, and their ability to bundle is required for biofilm structure.^6,22^ These fibers have been found to polymerize spontaneously *in vitro*(22) Due to their mode of polymerization, the addition of a single serine at the N-terminus blocks fiber formation yielding monomeric TasA.^23^ Recently, TasA was shown to associate with the lipid raft fraction of the cell membrane.^24−27^ Moreover, deletion of *tasA* decreased sliding motility on biofilm-inducing media but not on soft LB agar.^28^ In addition, TasA point mutants influence the hydrophobicity of the biofilm^27,29^ suggesting to us that TasA may also have interfacial activity. We set out to determine whether TasA, like BslA, is interfacially active and whether it influences the function of BslA at an interface.

## Materials and Methods

### Protein Production and Purification

Two versions of TasA were chosen to examine the properties of both the monomeric protein and the fibrous form (Figure 1C). The fibrous TasA construct included the mature secreted protein of residues 28–261 (fTasA), whereas the monomeric construct had an additional serine prior to residue 28 (mTasA).^23^ Recombinant BslA constructs used in this study included the secreted form lacking the unstructured N-terminus (hence comprising residues 42–181), termed BslA herein (Figure 1C). In some assays, a variant of this construct, in which cysteines 178 and 180 were mutated to alanine, was used (BslA AxA). This protein is unable to make covalent linkages and remains monomeric.^14^

BslA and TasA proteins were purified as previously described.^5,14,23^ In brief, for each protein, a 5 mL LB was supplemented with ampicillin (100 μg/mL) culture of *Escherichia coli* BL21 (DE3) pLysS cells containing the plasmid for overexpression of glutathione-S-transferase (GST)-BslA fusion, GST-BslA AxA fusion, GST-TasA fusion, or GST-mTasA fusion protein were grown overnight at 130 rpm in a 37 °C warm room (see Table S1 plasmid details). 1 L amount of autoinduction medium supplemented with ampicillin (100 μg/mL) was inoculated (1:1000 [vol/vol]) from the starter cultures and grown at 37 °C and 200 rpm until an OD_600_ of 0.9 was reached. Induction of protein expression was induced by reducing the temperature to 18 °C for further incubation overnight. Cells were harvested by centrifugation at 4000*g* for 30 min and stored at −80 °C until further use.

For purification, bacterial pellets were thawed and resuspended in a purification buffer (25 mM Tris-HCl pH 7.5, 250 mM NaCl for fTasA and mTasA, or 50 mM HEPES pH 7.5, 250 mM NaCl for BslA) supplemented with Complete EDTA-free Proteinase Inhibitors (Roche) before lysis using an Emulsiflex cell disruptor (Avestin). Cellular debris was separated by centrifugation at 27,000*g* for 30 min, and the resulting supernatant was incubated with Glutathione Sepharose 4B resin (GE Healthcare) agarose at a ratio of 750 μL resin per 1 L bacterial culture with gentle rotation for 2–4 h at 4 °C. The mixture of lysate plus beads was passed through a single-use 25 mL gravity flow column (Bio-Rad). The beads were washed twice with 20 mL of an appropriate purification buffer. The GST tag was removed by TEV protease treatment in 25 mL of purification buffer with 0.5 mg of TEV and 1 mM DTT. Following overnight cleavage at 4 °C, the cleaved protein was isolated using a clean gravity column. The TEV protease and any remaining GST was removed by adding 750 μL of fresh Glutathione Sepharose plus 250 μL of Ni-NTA agarose. The solution was incubated with gentle rotation overnight at 4 °C. A final gravity column step led to a flowthrough containing the clean, purified protein. The purified proteins were then concentrated and simultaneously buffer exchanged into 25 mM phosphate buffer pH 7.0 using Vivaspin 20 centrifugal concentrators. Protein purity was evaluated by sodium dodecyl sulfate–polyacrylamide gel electrophoresis (SDS–PAGE). The fTasA sample after purification is intrinsically heterogeneous containing a distribution of fibrils of variable lengths.^23^

### Emulsions

Emulsions were created by mixing an 8 mg/mL protein solution in pH 7 phosphate buffer (mTasA or fTasA) with glyceryl trioctanoate (GTO) in an 80:20 (v/v) ratio. The oil/water solution was mixed for 1 min using a rotor stator (IKA Ultra-Turrax T10) at a shear rate of 20,000 s^–1^. The emulsions were allowed to cream for 20 min after which a 10 μL sample was withdrawn (from the cream fraction if separation occurred) and diluted with phosphate buffer at a ratio of 1:50. This diluted sample was then placed in a cavity slide and covered with a coverslip, which was sealed with clear nail varnish (BarryM All-in-One) to prevent evaporation. Images of the emulsion droplets were captured by using a Nikon Ti–U inverted microscope outfitted with a 10× objective. The slides were kept at room temperature for 1 week and reimaged.

### Circular Dichroism Spectroscopy

Circular dichroism spectroscopy of mTasA in solution was performed by the Glasgow Structural Biology Biophysical Characterization Facility. Measurements were performed using a Jasco J-810 spectropolarimeter at an mTasA concentration of 0.5 mg/mL in a 0.02 cm quartz cuvette. Scans were performed in continuous mode between 260–200 nm at a speed of 10 nm/min. The data pitch was 0.2 nm, with a response time of 2 s. Three scans were accumulated and averaged to produce the final curve.

For fTasA, samples were measured at a protein concentration of 0.2 mg/mL (in 25 mM phosphate buffer) in a 0.1 cm quartz cuvette. A scan rate of 50 nm s^–1^ was used with a data pitch of 0.1 nm and a digital integration time of 1 s. Twenty scans were accumulated and averaged to produce the final curve.

RIMEs were made by first preparing an 80:20 (v/v) decane emulsion with 0.2 mg/mL of protein. The emulsion was mixed for 1 min using a rotor stator (IKA Ultra-Turrax T10) at a shear rate of 20,000 s^–1^. The emulsion was washed 3 times to remove any residual protein not adsorbed to the oil/water interface. Washes were performed by centrifuging at 1000 rpm for 20 s, a portion of the subphase was removed and replaced with buffer, then gently redispersed. Finally, the subphase was removed and replaced with glycerol to 59% (w/v), at which point the emulsion became transparent. The emulsion was gently remixed on a roller bank and then allowed to cream. The cream was placed in a 1 mm path length quartz cuvette for spectroscopy. To prevent creaming during the experiment, the cuvette was briefly inverted between measurements to redisperse the droplets.

### Pendant Drop Tensiometry

Pendant drop experiments were performed on an EasyDrop tensiometer (Krüss, Hamburg, Germany). Protein solutions were diluted to the desired concentration with phosphate buffer made from Milli-Q water and placed in a syringe with a 1.83 mm diameter needle. For the wrinkle relaxation experiments, a 30 μL aqueous droplet of protein solution was expelled into GTO and allowed to equilibrate at room temperature for 30 min. Images were acquired at 1 fps for BslA and 2 fps for mTasA by using a digital camera. Wrinkle relaxation experiments were performed following compression of the droplet, which was achieved by retracting a volume of the protein solution (10 μL, unless otherwise stated). Compression induced the formation of wrinkles in the surface layer. The wrinkles were monitored over a 10 min period. To analyze the relaxation of the wrinkles, a line profile was drawn across the wrinkles. The line profile was plotted using the grayscale values (from 0 to 255) of each pixel along this line using ImageJ. At least 10 wrinkles were monitored over the time course of the experiment. To plot the relaxation rate, the grayscale value of the pixels was normalized, and background corrected (Supporting Figure 1).

Measurements of the dynamic interfacial tension were determined by fitting the droplet shape to the Young–Laplace equation. The dynamics of interfacial protein adsorption can often be characterized by three kinetic regimes: Regime I is a “lag time” where there is no apparent change in the interfacial tension, Regime II is when a sufficient proportion of protein adsorbs to the interface to produce a decrease in interfacial tension, and Regime III is reached when the interfacial tension plateaus to a roughly constant final value. We define the time to interface as the transition between Regime I and Regime II. This was measured by fitting linear functions to Regime I and Regime II and finding their time of intersection.

### Brewster Angle Microscopy

BslA AxA (BslA 42–181 C178A C180A) was diluted to 0.005 mg/mL prior to the experiment. The solution was poured into a trough to allow the formation of a BslA single-layer film over time. The imaging area was roughly 2–3 cm away from the Wilhelmy plate. Equilibration was performed with the barriers fully withdrawn (*A* = 250 cm^2^). Sequential BAM imaging was performed using a Nanofilm E3Pse at the Diamond Light Source, U.K. In all images, black pixels represent the air/water interface, while nonblack pixels are representative of adsorbed interfacial material.

## Results and Discussion

### TasA Stabilizes Oil-in-Water Emulsions

**Figure 2:**
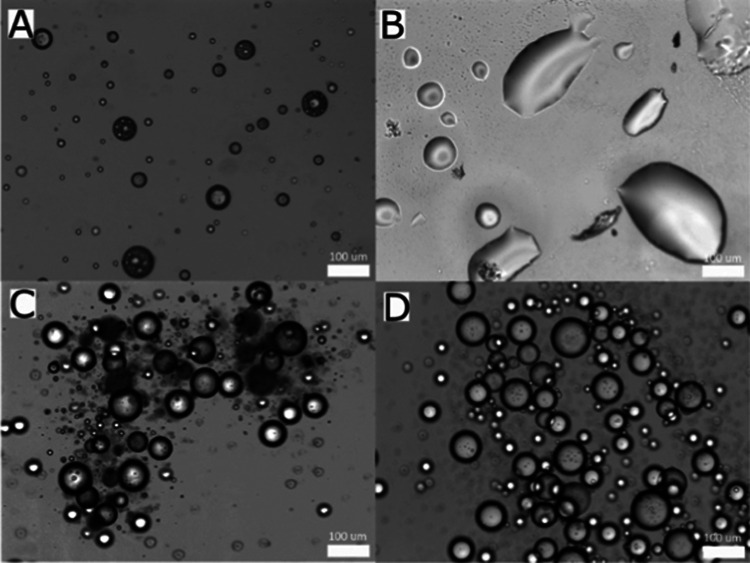
TasA stabilizes oil–water emulsions. Microscope images of oil–water–oil droplets produced from mixing 8 mg/mL mTasA (A, B) or fTasA (C, D) in phosphate buffer with GTO (80:20 v/v). Images are from two time points: immediately after emulsification (A, C) and after 1 week of incubation at room temperature (B, D). Scale bar is 100 μm.

We first wished to determine whether TasA can stabilize emulsions, as an indication of potential interfacial activity. To test this, we produced oil-in-water (O–W) emulsions in the presence of fTasA or mTasA and found that both forms of the protein could stabilize emulsion droplets (Figure 2A,C). The emulsions stabilized by mTasA were uniformly dispersed in the continuous phase, with some droplets forming multiple emulsions (W–O–W) (Figure 2A). In contrast, when emulsions were stabilized using fTasA, we found a mixture of dispersed and clustered droplets (Figure 2C), although within the population, we also observed multiple emulsions. The clustered droplets, when viewed under the microscope, appeared bound together or bridged into units that can span hundreds of micrometers in size. We monitored the stability of the emulsions over a span of 1 week and found that the emulsions stabilized by mTasA had largely coalesced (Figure 2B). In contrast, after 7 days, the emulsions stabilized by fTasA were structurally like those observed immediately after emulsification, and both the characteristic flocculation and presence of multiple emulsions could still be clearly observed (Figure 2D). In comparison, emulsions formed under the same conditions but stabilized by BslA were stable and unchanged over the time scale of weeks.^30^ These results show that both forms of TasA can stabilize emulsion droplets, indicating that TasA possesses some degree of interfacial activity.

### TasA Changes Structure at the Interface

**Figure 3:**
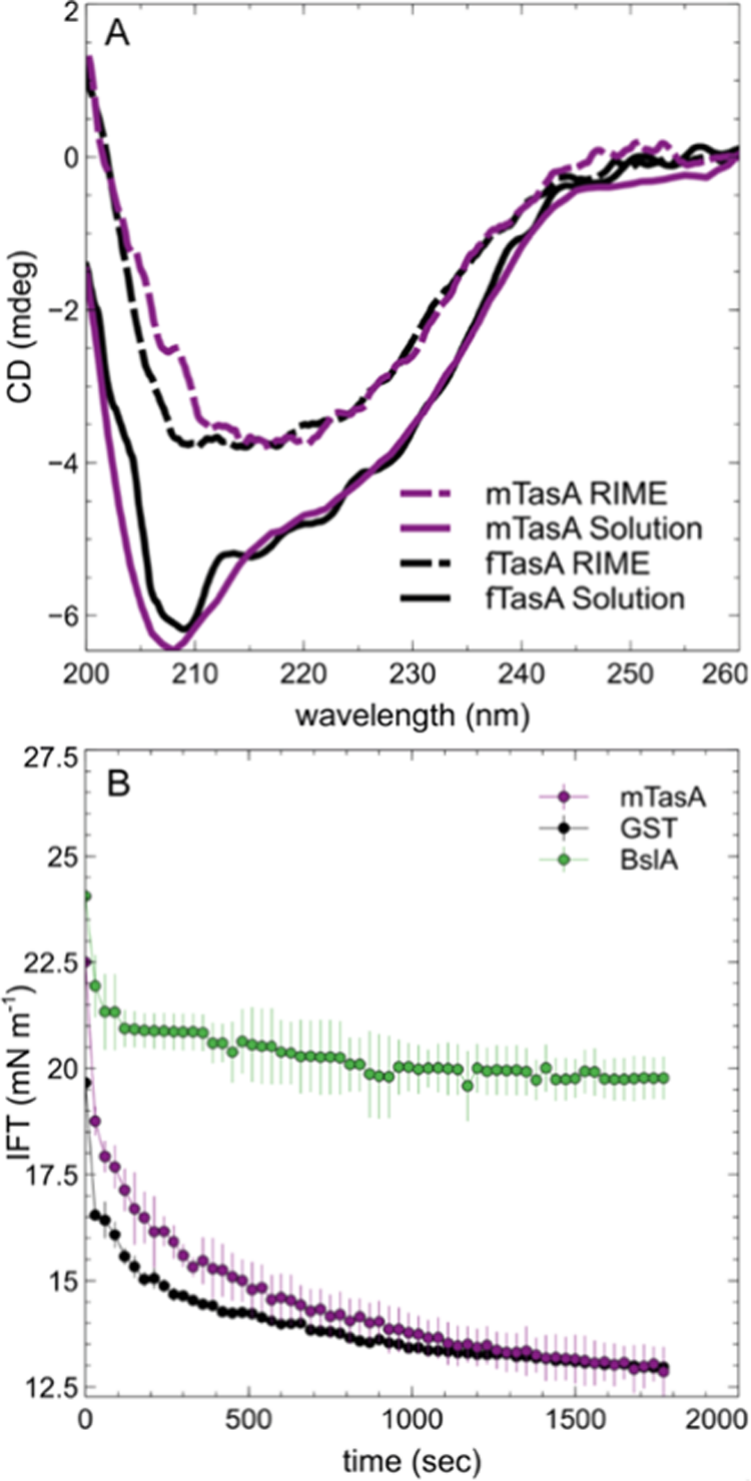
TasA undergoes structural changes upon adsorption to an interface. (A) CD spectroscopy of mTasA (purple) and fTasA (black) in solution (solid lines) and in RIMEs (dashed lines) shows a change in the secondary structure. (B) Pendant drop tensiometry reveals the time evolution of the interfacial tension (IFT) of a droplet of 0.1 mg/mL protein (mTasA, GST, and BslA) in GTO. The mean of three droplets is plotted for each protein with error bars representing SEM.

Many proteins exhibit interfacial activity; however, surface interactions can often result in partial or full denaturation of the protein structure.^31−37^ There are also, however, examples of natural protein biosurfactants that possess high surface activities while maintaining large-scale secondary and tertiary structures.^5,10,38,39^ Using circular dichroism spectroscopy (CD), we previously showed that BslA does not denature at an interface but instead undergoes a structural transition to a more β-sheet rich structure.^10^ To interrogate the structure of TasA at an oil/water interface, we compared the CD spectra of fTasA and mTasA when in an aqueous solution to the equivalent protein-stabilized emulsions. In solution, both forms of TasA possess similar CD spectra with mixed secondary structure content (Figure 3A). These results are consistent with the reported crystal and cryo-electron microscopy structures and previously published CD spectra of TasA.^19,21^ When fTasA and mTasA absorb at an oil/water interface, however, both exhibit a structural shift toward greater β-sheet content (Figure 3A). This could be a transition of α-helices or unstructured regions to β-sheets. These findings are reminiscent of the structural transition observed for BslA at an interface.^10^ Taken together, these results show that TasA does not denature at an interface but instead undergoes a structural transition, as in other known biosurfactants.

### TasA Adsorbs to the Interface

Samples of fTasA are inherently heterogeneous, varying in the average length of fiber and degrees of higher-order self-assembly into bundles, with a consequent impact on sample viscosity.^23^ Given that the CD spectroscopy results (Figure 3A) show that fTasA and mTasA have very similar secondary structures and undergo a similar structural change when adsorbed to an interface, we focus on the activity and properties of mTasA for the remainder of the study to remove the potential influence of sample heterogeneity. To further assess the interfacial activity of mTasA, we performed pendant drop tensiometry to monitor the kinetics of interfacial adsorption (Figure 3B). We compared mTasA to the eukaryotic detoxification enzyme Glutathione-S Transferase (GST), as a generic globular protein control with a molecular weight close to that of mTasA, and BslA as a known biosurfactant. We found that mTasA significantly lowers the interfacial tension (IFT) between the aqueous droplet and the continuous oil phase, reaching a value of ∼12.5 mN m^–1^ over a time scale of ∼30 min. Similarly, GST also saturates to an IFT of ∼12.5 mN m^–1^ (Figure 3B). In contrast, the initial decrease in IFT caused by the adsorption of BslA to the interface plateaus to a value of ∼20 mN m^–1^ (Figure 3B), as previously observed.^10,13^ This IFT “arrest” is due to the formation of a self-assembled elastic protein layer that no longer conforms to the Young–Laplace relation. These results show that while TasA and BslA share similar secondary structural changes when interacting with an interface, the kinetic characteristics of mTasA interfacial adsorption are closer to those of a generic globular protein than to BslA.

### TasA Affects BslA Film Formation *via* Competition

In view of the previous results, we wished to understand whether there is competition or interaction between BslA and TasA at an interface since they originate from the same biological system and are both produced during biofilm formation. First, we studied the change in the IFT as a function of time using pendant drop tensiometry when both BslA and TasA, or BslA and GST, were copresent in the aqueous phase. For both mixed samples, we did not observe an arrest at high IFT values as seen with BslA alone (c.f. Figure 3B). Since this arrest is due to the formation of an elastic film, this result implies that there is no continuous elastic surface layer formed when there is a mixture of both BslA and other interfacially active proteins at the interface.

**Figure 4:**
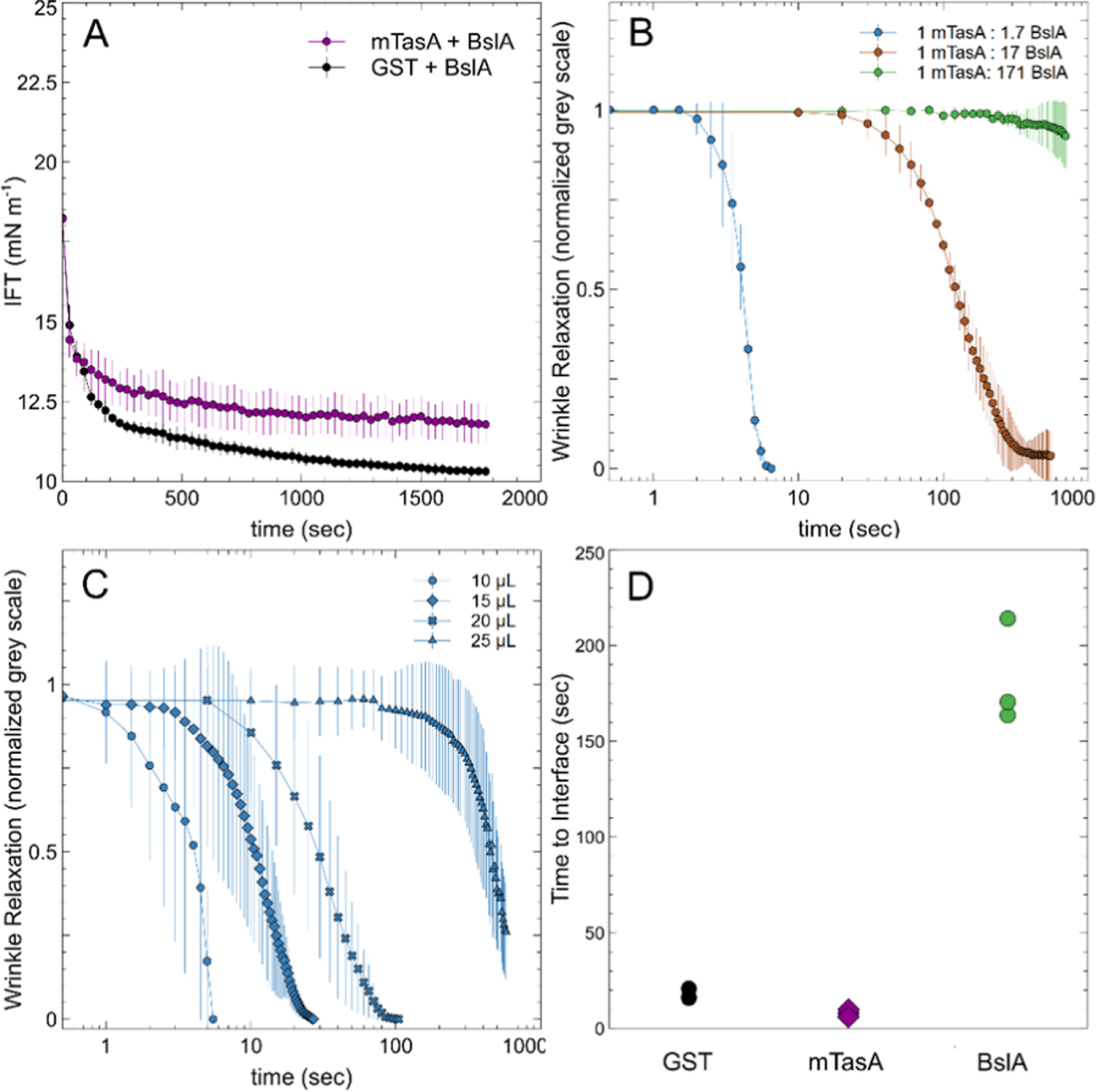
TasA affects the film formation of BslA. (A) Pendant drop tensiometry of BslA (0.2 mg/mL, 6.6 mM) with mTasA (0.1 mg/mL, 3.8 mM) or GST (0.1 mg/mL, 3.8 mM) at an oil/water interface shows a drop in the IFT over time. (B) Effect of mTasA on BslA film formation is dose-dependent as measured by wrinkle relaxation assays. Retraction of 10 μL from an equilibrium state 40 μL droplet in GTO led to visible wrinkles. The relaxation of wrinkles was plotted as a function of time for three different ratios of TasA to the BslA dimer. The concentration of BslA was the same as that in panel (A) at 0.2 mg/mL. (C) Wrinkle relaxation of mTasA/BslA mixture (1:1.7 molar ratio) plotted as a function of time for varied retraction volumes. (D) Time to interface calculated from pendant drop tensiometry for 0.03 mg/mL GST, mTasA, and BslA at an air–water interface for three independent experiments. All plots show the mean of three droplets with error bars representing SEM.

To explore this hypothesis further, we investigated the robustness of the BslA elastic film formed in the presence or absence of mTasA. An aqueous droplet containing protein was expelled into the oil phase on the end of a needle, and the protein adsorption was allowed to reach equilibrium (30 min). The droplet was then compressed by the retraction of a known volume of fluid back into the needle. Previous work has shown that BslA forms highly elastic films that wrinkle on droplet compression and that these wrinkles do not relax over time^5^ (Supporting Information (SI), Movie 1). In contrast, mTasA alone does not produce a wrinkled elastic layer upon compression (SI, Movie 2). To see how the BslA elastic layer is influenced by the presence of mTasA, we added increasing amounts of mTasA mixed with a constant concentration of BslA (0.2 mg/mL or 6.6 mM of BslA dimer) and performed the wrinkle relaxation experiments. We find that at high concentrations of mTasA (0.1 mg/mL or 3.8 mM) at a molar ratio of TasA to dimeric BslA of ∼1:1.7, an elastic layer is formed but relaxes very quickly (∼10 s) (Figure 4B). This implies that a BslA film can form in the presence of mTasA since mTasA on its own does not display the formation of wrinkles under compression. However, the presence of mTasA significantly weakens the robustness of the BslA film at these concentrations. A 10-fold decrease in the mTasA concentration (0.38 mM, ratio of ∼1:17) resulted in a film relaxation time that was nearly 30 times longer (Figure 4B). Another 10-fold decrease in mTasA concentration (0.038 mM, ratio of ∼1:171) produced relaxation behavior that was longer than the experimental time window and resembled the relaxation behavior of BslA alone (Figure 4B). These findings reveal a dose-dependency in which the BslA film has decreased stability with an increase in the presence of another interfacially active protein, mTasA. It is possible that the presence of mTasA at the interface prevents BslA from forming an interconnected network that would otherwise stabilize the surface of the droplet. Alternatively, mTasA may interact with BslA at the interface, modulating the film-forming ability of BslA.

To further analyze TasA’s effects, we performed experiments where we studied the relaxation of mixed mTasA/BslA droplets with increasing retraction volumes (i.e., increasing compressions) at a constant molar ratio of 1:1.7. We find that by increasing the retraction volume, we observe films that have longer relaxation times (Figure 4C). At high droplet compressions (smaller droplet surface areas), BslA forms more robust films that maintain wrinkle formation. These results suggest that under compression, mTasA is desorbed from the interface while BslA remains. We propose that as the surface area is reduced, mTasA leaves the interface, allowing BslA to make more interactions with neighboring BslA molecules, thereby permitting the establishment of a space-spanning network that is more robust to mechanical perturbations. Since the concentration of mTasA in solution does not change, this result also suggests that mTasA does not modulate BslA self-assembly and film formation *via* protein–protein interactions.

Finally, we wished to understand how mTasA and BslA potentially compete for space at an interface. We measured the time to the interface for the two proteins using pendant drop tensiometry, this time at an air–water interface. Here, we define the time to interface as the transition time between Regime I and Regime II in the kinetics of interfacial adsorption. We find that BslA is approximately an order of magnitude slower adsorbing to the interface as compared to mTasA and also GST as a control protein (Figure 4D). This very rapid adsorption by mTasA and GST explains why the IFT decreases significantly in the mixed samples containing BslA (Figure 4A): mTasA and GST get to the interface first and dominate the kinetics. This finding also provides insight into why mTasA at high concentrations impacts the film stability, as seen in the mixed ratio pendant drop wrinkle relaxation (Figure 4B). In the wrinkle relaxation assay, pressure is applied after the proteins at the interface have reached an equilibrium state. The kinetics occurring at the beginning lead to a certain ratio of BslA to mTasA at the interface, with mTasA reaching the interface more quickly. Based on the emulsions which are stable for long periods, we suggest that TasA stably associates until the retraction of the droplet and the compressive force induces desorption.

### Evolution of BslA Film Formation as Revealed by Brewster Angle Microscopy

**Figure 5:**
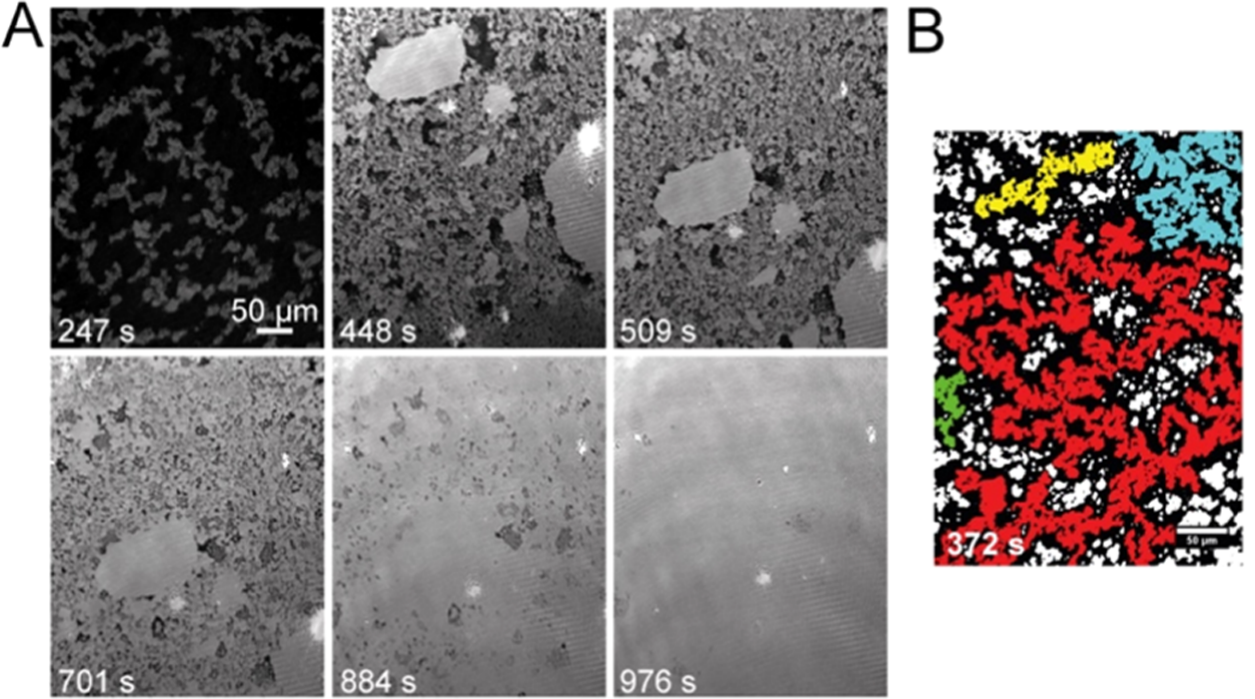
BslA film formation viewed by Brewster angle microscopy. (A) Images of a single region of the buffer/air interface over time labeled in seconds (s). Black pixels represent solution, and brighter pixels are interfacial material (0.005 mg/mL BslA protein). The first image at 247 s shows the microdomains forming. Then clear islands become visible that migrate across the field of view 448 and 509 s. The last 3 time points show the filling of the film into a monolayer. (B) Network of BslA film domains *t* = 372 s with each large continuous region given a unique color (e.g., red, cyan, yellow, and green) to highlight the extent of interconnectivity. The image was binarized after the threshold greyscale value of 12 was set. All scale bars are 50 μm.

To provide additional insight into the results above, we investigated the structure and evolution over time of BslA film formation at an interface using Brewster Angle Microscopy (BAM). To ensure a monolayer of protein at the interface, we employed BslA AxA, which is monomeric in solution.^14^ In this experiment, sequential BAM images were taken, and the resultant morphology of the surface layer was imaged (Figure 5A). Small anisotropic clusters of micrometer-scale domains were prevalent at the early stages of film development. From ∼350 s onward and a surface coverage of ∼51%, we observed continuously connected regions of BslA (Figure 5B). This was followed by the formation of large, smooth rafts or islands. It was also seen that movement of surface material slowed down until it essentially stopped by ∼500 s. Finally, we observe the formation of a continuous BslA layer at ∼1000 s within the field of view. We measured surface pressures during equilibration and during compression experiments, which indicated that a continuous layer across the entire trough was not completely achieved after this time frame (Supporting Figure 3). However, after only an area reduction of 15 cm^2^, we observed a sharp rise in surface pressure, which indicates a large surface layer was present after the initial equilibration. Taken together, these results show that BslA forms distinct, interconnected regions at early times, which grow to form larger rafts that ultimately results in a continuous protein layer at the interface. We additionally performed experiments using the pendant drop to test the film robustness as a function of droplet equilibration time. After a set equilibration time, the pendant drop volume was retracted to provide a compressive force, and we observed whether interfacial layer wrinkling occurred and, if so, how long those wrinkles persisted (Supporting Figure 1). We found that no wrinkling occurred for equilibration times up to 60 s. After 180 s equilibration times, wrinkles do form but relax within ∼40 s. We found longer persisting wrinkles that relax more slowly after 300 s equilibration. Finally, we found that after 600 s of equilibration, wrinkles persisted for the entirety of the observation window (10 min). Although the experimental conditions between trough and pendant drop experiments are different (e.g., protein concentration/surface area and hydrophobic phase), the time scales for film formation and film robustness as probed by pendant drop compression are broadly coincident.

Biofilms are biological communities of microorganisms formed by the secretion of materials that bind the collective together. In *B. subtilis* biofilms, TasA plays multiple roles, including structuring and maturation of the biofilm.^6^ TasA was also found to affect cellular physiology upon biofilm induction,^25^ and part of this was linked to its association with the detergent resistance fraction of the bacterial membrane.^27^ The multifunctionality, its location in the extracellular milieux, and its association with a membrane fraction led us to investigate whether TasA had an interfacial activity. Here, we have shown that the *B. subtilis* biofilm matrix proteins BslA and TasA both have interfacial activity but have very different biophysical properties.

Our results demonstrate that both monomeric and fiber forms of TasA can stabilize oil-in-water emulsion droplets. Interestingly, fTasA stabilized emulsions for significantly longer than the monomeric form. There are a few possible reasons for this. The fibrous form of TasA is more resistant to proteolysis and degradation than mTasA,^23^ and hence, intrinsic properties of the proteins could cause the variation in emulsion lifetime. Another possibility is that the multimeric structure affects the stability and/or kinetics of interfacial binding. The fibrous form is likely to create multivalent interactions with the interface. Thus, dissociation of one interfacially active unit (monomer) from the interface could occur, but dissociation of the whole fiber is unlikely. Dissociated units would also be held close to the interface and not diffuse away, leading to increased reassociation events. Additionally, there is evidence that TasA filaments bundle, and this side-to-side interaction may further stabilize the protein at the interface. Another interesting difference between fTasA and nonfibrous mTasA is that fTasA emulsions are nonuniform with persistent clusters of droplets evident. This could be due to filament-filament bundling or bridging between droplets. Another possibility is that one filament participates in the stabilization of more than one droplet. Previous work studying protein fibril stabilization of emulsions found that fibrils above a critical concentration can act as a depletant, inducing flocculation.^40,41^ Other work studying Pickering emulsion stabilized by cellulose fibers found that three-dimensional (3D) fiber-flocculated droplet networks could be created. Network formation was dependent on fibril morphology, where highly bundled fibers induced flocculation *via* depletion, whereas well-separated fibrils created networks of emulsion droplets *via* interfacial adsorption and bridging.^42^ These networks were highly stable and are reminiscent of those observed for fTasA (Figure 1C). Indeed, the size and morphology of the cellulose fibrils are similar to fTasA.^23^ It is, therefore, plausible that such a mechanism is at play in the formation of the fTasa-stabilized emulsion networks.

Both multimerization states of the protein undergo a structural change at the interface with an increased β-sheet content. The recent cryo-EM structure of the TasA fiber and the crystal structure of the TasA monomer show very little structural difference between the two, which was also observed by CD previously^23^ and herein. This raises the question of which region of the protein undergoes rearrangement at the interface. From the structures, we found a cluster of three helices on the side of the β-sandwich that is not involved in filament formation. The helicity of this region is not predicted by secondary structure servers (JPred and Phyre2^43,44^), and the region has high B-factors in the crystal structure (indicative of variation between monomers in the crystal or motion of the region); thus, it may represent a region capable of structural rearrangement. This would explain the primary β-sheet minima found in the CD-RIMEs spectra for the interfacially associated protein.

Protein-interfacial interactions can often result in denaturation of the protein structure. However, this work has shown that TasA undergoes a similar restructuring as BslA when adsorbed to an interface.^10^ There are other instances of proteins that interact with interfaces that demonstrate this type of reorganization while retaining their overall secondary structure. Ranaspumin-2 (Rsn-2), a biosurfactant associated with the foam nests of the Túngara frog, was shown to undergo a dramatic “clam-shell-like” reorganization of its tertiary structure when adsorbed to an interface while retaining its secondary structure motifs.^38^ Moreover, it was found that this process was reversible: the protein could be removed from the interface through compressive forces and then return *via* diffusion to readsorb to the interface. A similar reversible restructuring has also been reported for the apolipoprotein apolipophorin-III produced by the insect *Galleria mellonella*.^45^ This structural plasticity and reversibility of adsorption would be advantageous to the organism, as it permits the versatile use of protein resources. For instance, we have shown that BslA has a multipurpose function beyond simply forming a hydrophobic layer at the exterior of the biofilm.^14^ Similarly, the reversibility of apolipophorin-III allows the organism to reuse this resource for lipoprotein transport, lipopolysaccharide detoxification, and pattern recognition during an innate immune response.^46^ We suggest that TasA may also fall within this class of reusable or multipurpose biosurfactants.

The relatively longer TTI of BslA has been attributed to the necessity for the protein to restructure at or prior to reaching the interface. Indeed, point mutants to the BslA “cap” caused changes to the TTI supporting dependency on the cap restructuring.^13^ The TTI of mTasA is much shorter than that of BslA, suggesting a lower barrier to adsorption despite also going through restructuring (Figure 4D). It is likely that many factors affect adsorption dynamics, causing the differences between BslA and TasA TTIs, including surface hydrophobicity, the area that restructures, and the energetic barrier to restructuring in solution.

When BslA is adsorbed to the interface, it forms a robust elastic layer that dissipates the energy of compression through deformation or wrinkling. These deformations last indefinitely, contrasting sharply with the TasA films, which form transient wrinkles that very rapidly dissipate. This behavior is like that observed for the biosurfactant Rsn-2 and implies that TasA is removable from an interface when compressive forces are applied.^38^ Further work is required to investigate the reversibility of not only monomeric but also fibrous TasA adsorption to an interface, given that fibrous TasA appears to make multivalent interactions.

We have shown that the coadsorption of BslA with other proteins can have a marked effect on both the interfacial tension as well as the ability to form a strong elastic layer. Moreover, our results show that BslA is outcompeted with the interface: BslA is significantly slower to adsorb to the interface compared to TasA and our control protein GST, which prevents BslA from making a strong film. This observation suggests that BslA lateral interactions are integral to the stability of the film. In concert with the BAM results, we posit that the adsorption of other proteins is likely to impede or disrupt the time evolution of BslA network formation. The data in Figure 4B can then be understood within this context: more TasA will take up more space at the interface, interfering with the ability of BslA to form a space-spanning film. Therefore, at those higher TasA concentrations, we observe modified film relaxation dynamics. Moreover, the data from Figure 4C can be rationalized within this model. Under compression, mTasA returns to the aqueous phase, while BslA remains. At greater volume reductions, more mTasA is eliminated from the interface, allowing more contact between BslA molecules and resulting in greater film stability. Similar behavior was observed for BslA when mixed emulsions were made with casein, wherein BslA maintained stable emulsions despite cooling, Tween-80 addition, and melting.^30^

In view of these results, a question arises: how does BslA form a hydrophobic layer at the surface of a biofilm *in vivo* when there are a multitude of other molecular components possibly interacting with and competing at the air/water interface? Our pendant drop results (Figure 4) show that some film rigidity is still possible when BslA is coadsorbed to an interface. Importantly, compressive forces enhance BslA film formation since BslA remains so tightly bound to the interface relative to other proteins. We speculate that compressive forces within the biofilm (driven by e.g., evaporation and cellular growth) favor the retention of BslA at the interface over other surface-active molecules. Indeed, the highly wrinkled phenotype of *B. subtilis* biofilms shows that many compressive forces are at work within a growing biofilm, which could aid in the establishment of robust BslA surface layers.

## Conclusions

In this work, we explored the interfacial behavior of two primary protein matrix components of *B. subtilis* biofilms. We demonstrated that the protein TasA stabilizes oil-in water emulsions and that the fibrillar form of the protein exhibits long-term emulsion stability versus the monomer. We showed that the adsorption of TasA to an interface does not result in the denaturation of the protein but rather induces a partial conformational change with an enhancement of β-sheet content. We further investigated the dynamics of TasA interfacial adsorption using pendant drop tensiometry and found that TasA rapidly adsorbs to an interface but does not form an elastic film. This behavior contrasts with the other important biofilm matrix component, BslA, which adsorbs to the interface an order magnitude slower than TasA but forms a robust elastic layer. The stability and mechanical properties of the BslA film could be modulated through the presence and coadsorption of TasA. We performed Brewster Angle Microscopy and imaged the dynamical development and morphological characteristics of the BslA layer, which showed that the protein forms an interconnected gel-like network before forming a continuous layer at an air–water interface. These observations pose new questions about how BslA and TasA may function in an *in vivo* setting as well as opening new approaches to control the structure and mechanical properties of interfacial protein films.

## Supporting Information Available

Plasmids used in this study; diagram of wrinkle analysis; air/water interface kinetics; BAM surface pressure isotherms, and film relaxation dynamics as a function of equilibration time (PDF)
BslA forms a robust interfacial film. A 40 L droplet of 0.1 mg/mL BslA is expelled into GTO after 30 min of equilibration time. 10 L volume is withdrawn and subsequently, long-lived wrinkles form within the elastic BslA interfacial layer. The frame rate is 0.1 frame/s (AVI)
mTasA does not form a robust interfacial film. A 40 L droplet of 0.1 mg/mL mTasA is expelled into GTO after 30 min of equilibration time. 10 L volume is withdrawn, and it is observed that there is no wrinkling. Additional volumes are withdrawn until the droplet is very small, at which time a very transiently wrinkled film is observed. The frame rate is 1 frames/s (AVI)

## Supplementary Material

la3c03163_si_001.pdf

la3c03163_si_002.avi

la3c03163_si_003.avi

## Abbreviations

CD: circular dichroism
IFT: interfacial tension
BAM: Brewster angle microscopy

## References

[ref1] Hall-Stoodley L.; Costerton J. W.; Stoodley P. Bacterial Biofilms: From the Natural Environment to Infectious Diseases. Nat. Rev. Microbiol. 2004, 2 (2), 95–108. 10.1038/nrmicro821. 15040259

[ref2] Flemming H.-C.; Wingender J. The Biofilm Matrix. Nat. Rev. Microbiol. 2010, 8 (9), 623–633. 10.1038/nrmicro2415. 20676145

[ref3] Hobley L.; Harkins C.; MacPhee C. E.; Stanley-Wall N. R. Giving Structure to the Biofilm Matrix: An Overview of Individual Strategies and Emerging Common Themes. FEMS Microbiol. Rev. 2015, 39 (5), 649–669. 10.1093/femsre/fuv015. 25907113 PMC4551309

[ref4] Kobayashi K.; Iwano M. BslA (YuaB) Forms a Hydrophobic Layer on the Surface of Bacillus Subtilis Biofilms. Mol. Microbiol. 2012, 85 (1), 51–66. 10.1111/j.1365-2958.2012.08094.x. 22571672

[ref5] Hobley L.; Ostrowski A.; Rao F. V.; Bromley K. M.; Porter M.; Prescott A. R.; MacPhee C. E.; Van Aalten D. M. F.; Stanley-Wall N. R. BslA Is a Self-Assembling Bacterial Hydrophobin That Coats the Bacillus Subtilis Biofilm. Proc. Natl. Acad. Sci. U.S.A. 2013, 110 (33), 13600–13605. 10.1073/pnas.1306390110. 23904481 PMC3746881

[ref6] Branda S. S.; Chu F.; Kearns D. B.; Losick R.; Kolter R. A Major Protein Component of the Bacillus Subtilis Biofilm Matrix. Mol. Microbiol. 2006, 59 (4), 1229–1238. 10.1111/j.1365-2958.2005.05020.x. 16430696

[ref7] Arnaouteli S.; MacPhee C. E.; Stanley-Wall N. R. Just in Case It Rains: Building a Hydrophobic Biofilm the Bacillus Subtilis Way. Curr. Opin. Microbiol. 2016, 34, 7–12. 10.1016/j.mib.2016.07.012. 27458867

[ref8] Hölscher T.; Kovács Á. T. Sliding on the Surface: Bacterial Spreading without an Active Motor. Environ. Microbiol. 2017, 19 (7), 2537–2545. 10.1111/1462-2920.13741. 28370801

[ref9] Grau R. R.; de Oña P.; Kunert M.; Leñini C.; Gallegos-Monterrosa R.; Mhatre E.; Vileta D.; Donato V.; Hölscher T.; Boland W.; et al. A Duo of Potassium-Responsive Histidine Kinases Govern the Multicellular Destiny of Bacillus Subtilis. mBio 2015, 6 (4), 10–1128. 10.1128/mBio.00581-15. PMC4495169 26152584

[ref10] Bromley K. M.; Morris R. J.; Hobley L.; Brandani G.; Gillespie R. M. C.; McCluskey M.; Zachariae U.; Marenduzzo D.; Stanley-Wall N. R.; MacPhee C. E. Interfacial Self-Assembly of a Bacterial Hydrophobin. Proc. Natl. Acad. Sci. U.S.A. 2015, 112 (17), 5419–5424. 10.1073/pnas.1419016112. 25870300 PMC4418867

[ref11] Brandani G. B.; Schor M.; Morris R.; Stanley-Wall N.; MacPhee C. E.; Marenduzzo D.; Zachariae U. The Bacterial Hydrophobin BslA Is a Switchable Ellipsoidal Janus Nanocolloid. Langmuir 2015, 31 (42), 11558–11563. 10.1021/acs.langmuir.5b02347. 26378478

[ref12] Morris R. J.; Bromley K. M.; Stanley-Wall N.; MacPhee C. E. A Phenomenological Description of BslA Assemblies across Multiple Length Scales. Philos. Trans. R. Soc., A 2016, 374 (2072), 20150131 10.1098/rsta.2015.0131. PMC4920280 27298433

[ref13] Morris R. J.; Schor M.; Gillespie R. M. C.; Ferreira A. S.; Baldauf L.; Earl C.; Ostrowski A.; Hobley L.; Bromley K. M.; Sukhodub T.; et al. Natural Variations in the Biofilm-Associated Protein BslA from the Genus Bacillus. Sci. Rep. 2017, 7 (1), 6730 10.1038/s41598-017-06786-9. 28751732 PMC5532214

[ref14] Arnaouteli S.; Ferreira A. S.; Schor M.; Morris R. J.; Bromley K. M.; Jo J.; Cortez K. L.; Sukhodub T.; Prescott A. R.; Dietrich L. E. P.; et al. Bifunctionality of a Biofilm Matrix Protein Controlled by Redox State. Proc. Natl. Acad. Sci. U.S.A. 2017, 114 (30), E6184–E6191. 10.1073/pnas.1707687114. 28698374 PMC5544334

[ref15] Stanley-Wall N. R.; MacPhee C. E. Connecting the Dots between Bacterial Biofilms and Ice Cream. Phys. Biol. 2015, 12 (6), 063001 10.1088/1478-3975/12/6/063001. 26685107

[ref16] Kaufman G.; Liu W.; Williams D. M.; Choo Y.; Gopinadhan M.; Samudrala N.; Sarfati R.; Yan E. C. Y.; Regan L.; Osuji C. O. Flat Drops, Elastic Sheets, and Microcapsules by Interfacial Assembly of a Bacterial Biofilm Protein, BslA. Langmuir 2017, 33 (47), 13590–13597. 10.1021/acs.langmuir.7b03226. 29094950

[ref17] Bromley K. M.; MacPhee C. E. BslA-Stabilized Emulsion Droplets with Designed Microstructure. Interface Focus 2017, 7 (4), 20160124 10.1098/rsfs.2016.0124. 28630671 PMC5474033

[ref18] Schloss A. C.; Liu W.; Williams D. M.; Kaufman G.; Hendrickson H. P.; Rudshteyn B.; Fu L.; Wang H.; Batista V. S.; Osuji C.; et al. Fabrication of Modularly Functionalizable Microcapsules Using Protein-Based Technologies. ACS Biomater. Sci. Eng. 2016, 2 (11), 1856–1861. 10.1021/acsbiomaterials.6b00447. 29805990 PMC5967246

[ref19] Diehl A.; Roske Y.; Ball L.; Chowdhury A.; Hiller M.; Molière N.; Kramer R.; Stöppler D.; Worth C. L.; Schlegel B.; et al. Structural Changes of TasA in Biofilm Formation of Bacillus Subtilis. Proc. Natl. Acad. Sci. U.S.A. 2018, 115 (13), 3237–3242. 10.1073/pnas.1718102115. 29531041 PMC5879678

[ref20] Stöver A. G.; Driks A. Secretion, Localization, and Antibacterial Activity of TasA, a Bacillus Subtilis Spore-Associated Protein. J. Bacteriol. 1999, 181 (5), 1664–1672. 10.1128/JB.181.5.1664-1672.1999. 10049401 PMC93559

[ref21] Böhning J.; Ghrayeb M.; Pedebos C.; Abbas D. K.; Khalid S.; Chai L.; Bharat T. A. M. Donor-Strand Exchange Drives Assembly of the TasA Scaffold in Bacillus Subtilis Biofilms. Nat. Commun. 2022, 13 (1), 7082 10.1038/s41467-022-34700-z. 36400765 PMC9674648

[ref22] Romero D.; Aguilar C.; Losick R.; Kolter R. Amyloid Fibers Provide Structural Integrity to Bacillus Subtilis Biofilms. Proc. Natl. Acad. Sci. U.S.A. 2010, 107 (5), 2230–2234. 10.1073/pnas.0910560107. 20080671 PMC2836674

[ref23] Erskine E.; Morris R. J.; Schor M.; Earl C.; Gillespie R. M. C.; Bromley K. M.; Sukhodub T.; Clark L.; Fyfe P. K.; Serpell L. C.; et al. Formation of Functional, Non-amyloidogenic Fibres by Recombinant Bacillus Subtilis TasA. Mol. Microbiol. 2018, 110 (6), 897–913. 10.1111/mmi.13985. 29802781 PMC6334530

[ref24] Malishev R.; Abbasi R.; Jelinek R.; Chai L. Bacterial Model Membranes Reshape Fibrillation of a Functional Amyloid Protein. Biochemistry 2018, 57 (35), 5230–5238. 10.1021/acs.biochem.8b00002. 29565118

[ref25] Mielich-Süss B.; Schneider J.; Lopez D. Overproduction of Flotillin Influences Cell Differentiation and Shape in Bacillus Subtilis. mBio 2013, 4 (6), e00719-13 10.1128/mBio.00719-13. 24222488 PMC3892786

[ref26] López D.; Kolter R. Functional Microdomains in Bacterial Membranes. Genes Dev. 2010, 24 (17), 1893–1902. 10.1101/gad.1945010. 20713508 PMC2932971

[ref27] Cámara-Almirón J.; Navarro Y.; Díaz-Martínez L.; Magno-Pérez-Bryan M. C.; Molina-Santiago C.; Pearson J. R.; de Vicente A.; Pérez-García A.; Romero D. Dual Functionality of the Amyloid Protein TasA in Bacillus Physiology and Fitness on the Phylloplane. Nat. Commun. 2020, 11 (1), 1859 10.1038/s41467-020-15758-z. 32313019 PMC7171179

[ref28] Van Gestel J.; Vlamakis H.; Kolter R. From Cell Differentiation to Cell Collectives: Bacillus Subtilis Uses Division of Labor to Migrate. PLoS Biol. 2015, 13 (4), e1002141 10.1371/journal.pbio.1002141. 25894589 PMC4403855

[ref29] Dragoš A.; Martin M.; Falcón García C.; Kricks L.; Pausch P.; Heimerl T.; Bálint B.; Maróti G.; Bange G.; López D.; et al. Collapse of Genetic Division of Labour and Evolution of Autonomy in Pellicle Biofilms. Nat. Microbiol. 2018, 3 (12), 1451–1460. 10.1038/s41564-018-0263-y. 30297741

[ref30] MacPhee C.; Stanley-wall N.; Bromley K.; Morris R.; Hobley L. Synthetic Multiphase Systems, Google Patents, 2020.

[ref31] Clarkson J. R.; Cui Z. F.; Darton R. C. Protein Denaturation in Foam: I. Mechanism Study. J. Colloid Interface Sci. 1999, 215 (2), 323–332. 10.1006/jcis.1999.6255. 10419667

[ref32] Graham D. E.; Phillips M. C. Proteins at Liquid Interfaces: I. Kinetics of Adsorption and Surface Denaturation. J. Colloid Interface Sci. 1979, 70 (3), 403–414. 10.1016/0021-9797(79)90048-1.

[ref33] Jungbauer A.; Machold C.; Hahn R. Hydrophobic Interaction Chromatography of Proteins: III. Unfolding of Proteins upon Adsorption. J. Chromatogr. A 2005, 1079 (1–2), 221–228. 10.1016/j.chroma.2005.04.002. 16038308

[ref34] Tronin A.; Dubrovsky T.; Dubrovskaya S.; Radicchi G.; Nicolini C. Role of Protein Unfolding in Monolayer Formation on Air– Water Interface. Langmuir 1996, 12 (13), 3272–3275. 10.1021/la950879+.

[ref35] Yano Y. F. Kinetics of Protein Unfolding at Interfaces. J. Phys.: Condens. Matter 2012, 24 (50), 503101 10.1088/0953-8984/24/50/503101. 23164927

[ref36] Dickinson E.; Matsumura Y. Proteins at Liquid Interfaces: Role of the Molten Globule State. Colloids Surf., B 1994, 3 (1–2), 1–17. 10.1016/0927-7765(93)01116-9.

[ref37] Husband F. A.; Garrood M. J.; Mackie A. R.; Burnett G. R.; Wilde P. J. Adsorbed Protein Secondary and Tertiary Structures by Circular Dichroism and Infrared Spectroscopy with Refractive Index Matched Emulsions. J. Agric. Food Chem. 2001, 49 (2), 859–866. 10.1021/jf000688z. 11262041

[ref38] Morris R. J.; Brandani G. B.; Desai V.; Smith B. O.; Schor M.; MacPhee C. E. The Conformation of Interfacially Adsorbed Ranaspumin-2 Is an Arrested State on the Unfolding Pathway. Biophys. J. 2016, 111 (4), 732–742. 10.1016/j.bpj.2016.06.006. 27558717 PMC5002068

[ref39] Wösten H. A. B. Hydrophobins: Multipurpose Proteins. Annu. Rev. Microbiol. 2001, 55 (1), 625–646. 10.1146/annurev.micro.55.1.625. 11544369

[ref40] Blijdenstein T. B. J.; Veerman C.; van der Linden E. Depletion– Flocculation in Oil-in-Water Emulsions Using Fibrillar Protein Assemblies. Langmuir 2004, 20 (12), 4881–4884. 10.1021/la0497447. 15984245

[ref41] Peng J.; Simon J. R.; Venema P.; Van Der Linden E. Protein Fibrils Induce Emulsion Stabilization. Langmuir 2016, 32 (9), 2164–2174. 10.1021/acs.langmuir.5b04341. 26882086

[ref42] Yuan T.; Zeng J.; Wang B.; Cheng Z.; Chen K. Pickering Emulsion Stabilized by Cellulosic Fibers: Morphological Properties-Interfacial Stabilization-Rheological Behavior Relationships. Carbohydr. Polym. 2021, 269, 118339 10.1016/j.carbpol.2021.118339. 34294348

[ref43] Kelley L. A.; Mezulis S.; Yates C. M.; Wass M. N.; Sternberg M. J. E. The Phyre2 Web Portal for Protein Modeling, Prediction and Analysis. Nat. Protoc. 2015, 10 (6), 845–858. 10.1038/nprot.2015.053. 25950237 PMC5298202

[ref44] Cole C.; Barber J. D.; Barton G. J. The Jpred 3 Secondary Structure Prediction Server. Nucleic Acids Res. 2008, 36, W197–W201. 10.1093/nar/gkn238. 18463136 PMC2447793

[ref45] Leon L. J.; Idangodage H.; Wan C.-P. L.; Weers P. M. M. Apolipophorin III: Lipopolysaccharide Binding Requires Helix Bundle Opening. Biochem. Biophys. Res. Commun. 2006, 348 (4), 1328–1333. 10.1016/j.bbrc.2006.07.199. 16919602 PMC1851894

[ref46] Whitten M. M. A.; Tew I. F.; Lee B. L.; Ratcliffe N. A. A Novel Role for an Insect Apolipoprotein (Apolipophorin III) in β-1, 3-Glucan Pattern Recognition and Cellular Encapsulation Reactions. J. Immunol. 2004, 172 (4), 2177–2185. 10.4049/jimmunol.172.4.2177. 14764684

