# Supplementary material for: Bacillus subtilis Matrix Protein TasA is Interfacially Active, but BslA Dominates Interfacial Film Properties
Source: Langmuir. 2024 Feb 14;40(8):4164–73. doi: 10.1021/acs.langmuir.3c03163 (PMC10905994; doi:10.1021/acs.langmuir.3c03163)
Supplement: Supplementary file 1 — la3c03163_si_001.pdf [file la3c03163_si_001.pdf]

Supplementary Material

The *Bacillus subtilis* matrix protein TasA is interfacially active, but BslA dominates interfacial film properties.

AUTHOR NAMES: , Ryan J. Morris (1,2), Natalie C. Bamford (2,3), Keith M. Bromley (1)[$], Elliot Erskine (3)[%], Nicola R. Stanley-Wall (3), Cait E. MacPhee (1,2)*

AUTHOR ADDRESS: (1) School of Physics & Astronomy, University of Edinburgh, Peter Guthrie Tait Road, Edinburgh, EH9 3FD, United Kingdom. (2) National Biofilms Innovation Centre. (3) Division of Molecular Microbiology, School of Life Sciences, University of Dundee, Dundee, DD1 5EH United Kingdom.

Table S1. Plasmids used in this study

Name	Description	Reference
pNW1128	pGEX-6P-1 -TEV-*bslA* (residues 42-182)	(1)

pNW1505	pGEX-6P-1 -TEV-*bslA* C178A C180A (residues 42-182)	(2)
pNW1437	pGEX-6P-1 -TEV-*tasA* (residues 29-261)	(3)
pNW1080	pGEX-6P-1 -TEV-Ser-*tasA* (residues 29-261)	(3)

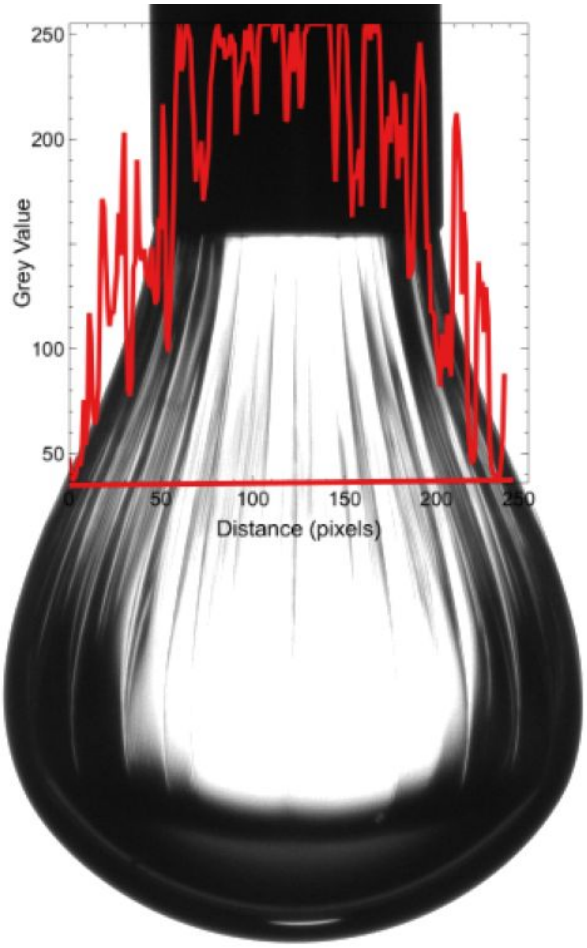

Supplemental Figure 1 Diagram of how film relaxation is measured. A line profile (red line across the middle of the droplet) is drawn and the grey values are obtained (red line on graph). The local minima of the profile are followed over time. The time that it takes for these minima to become equal to the background is taken to be the relaxation time.

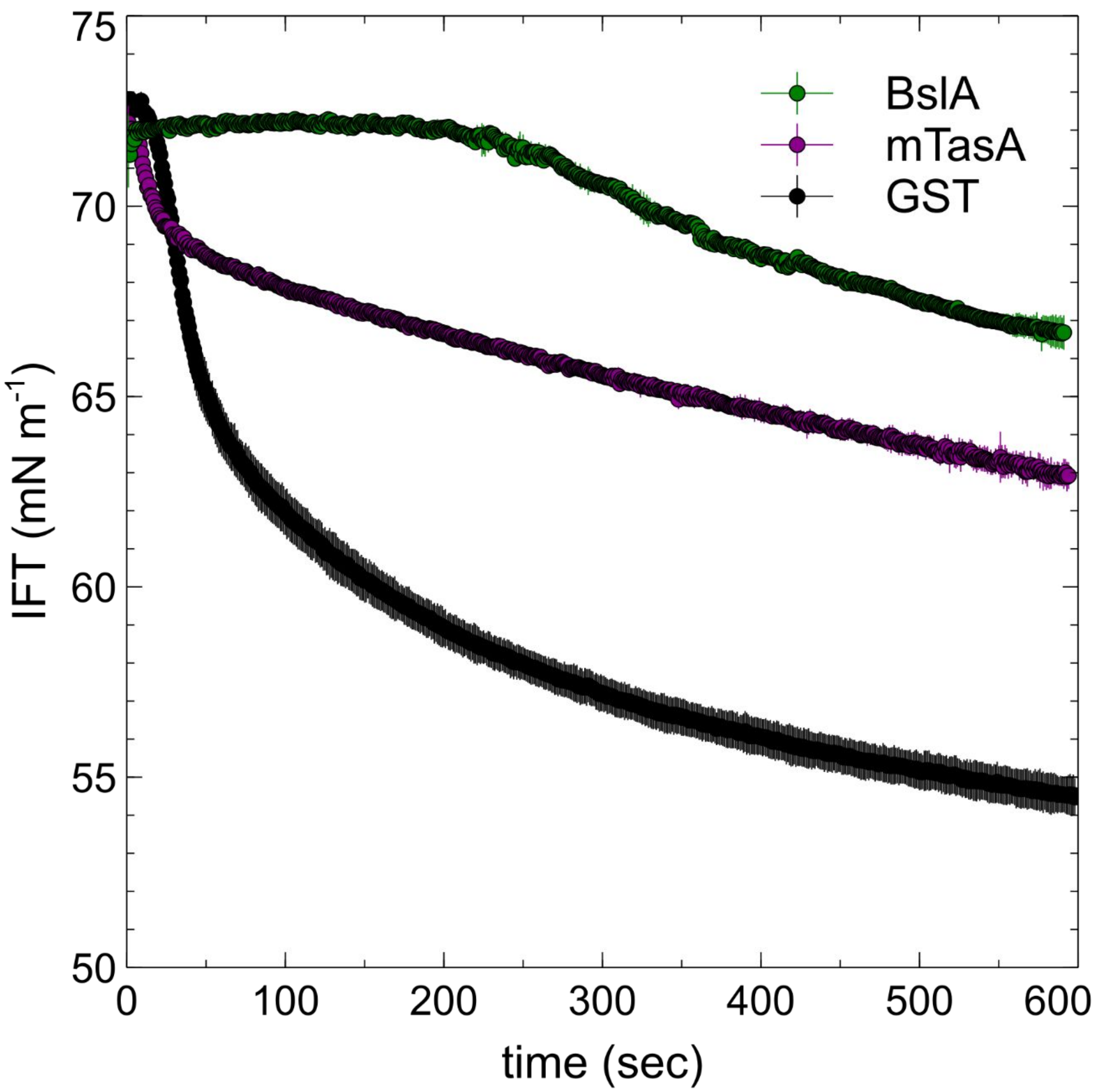

Supplemental Figure 2 IFT measurements of BslA, mTasA, and GST at an air-water interface.

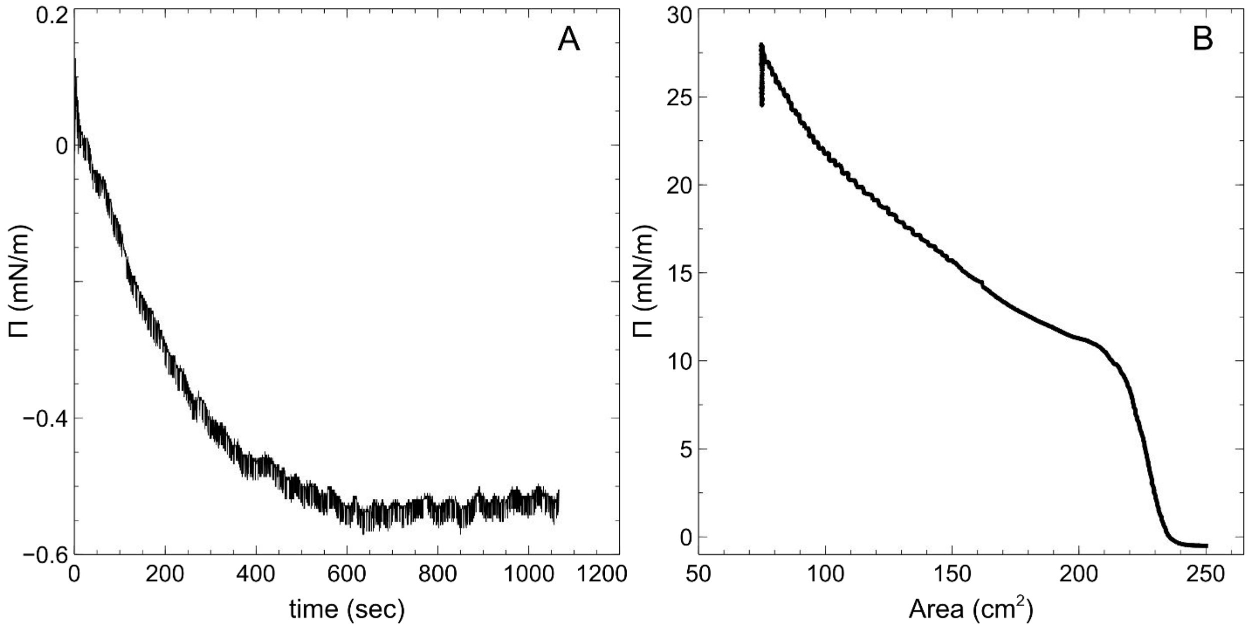

Supplemental Figure 3 Surface pressure measurement obtained during the BAM imaging in Fig. 5. (A) W observed an initial decrease in the surface pressure. The imaging revealed a continuous layer of protein within the field of view after 1000s. (B) Immediately after this equilibration we performed a compression experiment. An initial plateau in the surface pressure indicates that an entire network may not have been formed across the entire trough by this time point. However, surface pressures quickly rise until film buckling at approximately 70 cm^2.

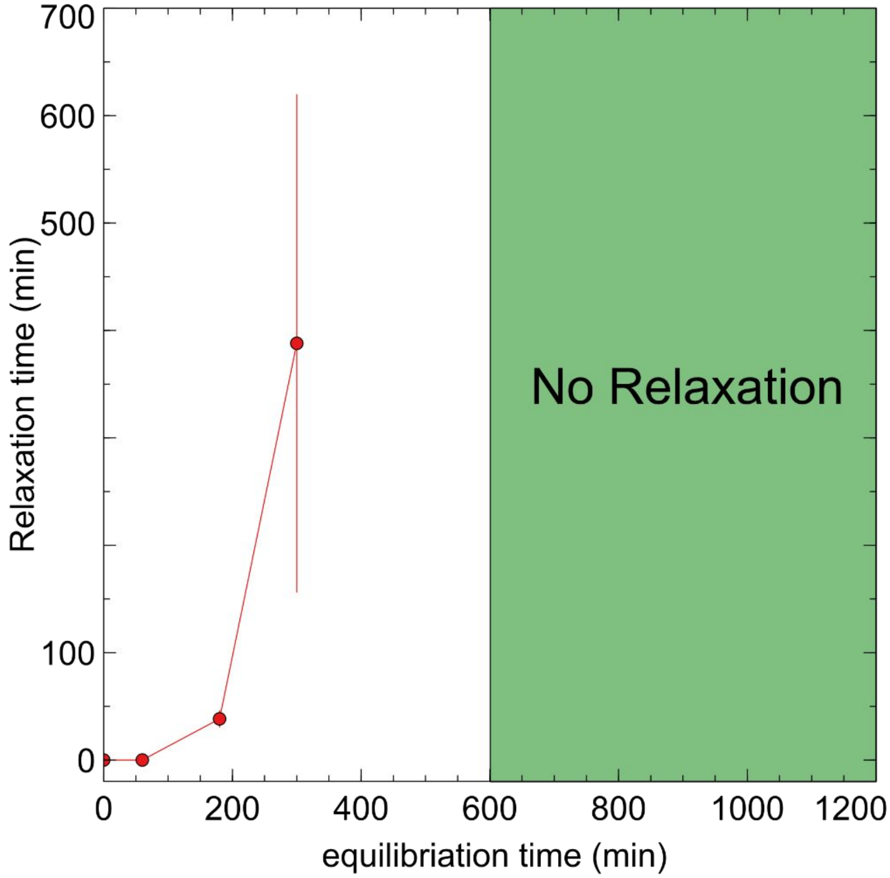
700
600
500
Relaxation time (min)
100
0
No Relaxation
0
200
400
600
800
1000
1200
equilibriation time (min)

Supplemental Figure 4. Film relaxation dynamics as a function of droplet equilibration time. A 40 μL droplet of 0.2 mg/ml BslA solution was expelled into GTO and allowed to equilibrate for 0, 1, 3, 5, 10, and 20 minutes. After the designated equilibration time, 10 μL volume was retracted to test probe the protein film properties. Three separate droplets for each equilibration time were imaged. Reported relaxation times correspond to when no wrinkles in the protein film are visible. For the 10- and 20-minute equilibration times, wrinkles persisted (green region) for the entire observation window (10 minutes). Relaxation times are the mean and standard deviation of the three experiments.

Supplemental Movie 1. BslA forms a robust interfacial film. A 40 μL droplet of 0.1 mg/ml BslA is expelled into GTO after 30 minutes of equilibration time. 10 μL volume is withdrawn and subsequently, long-lived wrinkles form within the elastic BslA interfacial layer. The frame rate is 0.1 frame/s.

Supplemental Movie 2. mTasA does not form a robust interfacial film. A 40 μL droplet of 0.1 mg/ml mTasA is expelled into GTO after 30 minutes of equilibration time. 10 μL volume is withdrawn, and it is observed that there is no wrinkling. Additional volumes are withdrawn until the droplet is very small, at which time a very transiently wrinkled film is observed. The frame rate is 1 frames/s.

Supplemental References

1) Hobley L, et al. (2013) BslA is a self-assembling bacterial hydrophobin that coats the Bacillus subtilis biofilm. Proc Natl Acad Sci U S A 110:13600-13605.
2) Arnaouteli, S.; Ferreira, A. S.; Schor, M.; Morris, R. J.; Bromley, K. M.; Jo, J.; Cortez, K. L.; Sukhodub, T.; Prescott, A. R.; Dietrich, L. E. P. Bifunctionality of a Biofilm Matrix Protein Controlled by Redox State. *Proceedings of the National Academy of Sciences* **2017**, *114* (30), E6184–E6191.
3) Erskine, E.; Morris, R. J.; Schor, M.; Earl, C.; Gillespie, R. M. C.; Bromley, K. M.; Sukhodub, T.; Clark, L.; Fyfe, P. K.; Serpell, L. C. Formation of Functional, Non-amyloidogenic Fibres by Recombinant Bacillus Subtilis TasA. *Mol Microbiol* **2018**, *110* (6), 897–913.
